# Supplementary material for: High prevalence of fecal carriage of Extended-spectrum beta-lactamase and carbapenemase-producing Enterobacteriaceae among food handlers at the University of Gondar, Northwest Ethiopia
Source: PLoS One. 2022 Mar 17;17(3):e0264818. doi: 10.1371/journal.pone.0264818 (PMC8929611; doi:10.1371/journal.pone.0264818)
Supplement: S1 File — (RTF) [file pone.0264818.s001.rtf]

Annex 9.6. Questionnaire English version 
Questionnaires to collect demographic data associated factors and antimicrobial susceptibility patterns of Extended-spectrum Beta-lactamase and carbapenemase-producing Enterobacteriaceae among food handlers at university of Gondar northwest, Ethiopia.
Identification 
Questionnaire number/Code________________ Name of interviewer __________                                        
Date of interview                  /              2021
Section I: General information on socio-demographic characteristics of the food handlers
No.	Questions 	Classification and Category Code  	Remark	
1.1	Gender	1. Male                    2. Female          		
1.2	Age	……….yrs		
1.3	Level of education	1. Illiterate             
2. Primary completed
3. Secondary completed and above		
1.4	Monthly income 	---------Ethiopian Birr		
1.5	Marital status 	1. Single   2. Married    
		
1.6	Family size	-------		
1.7	Service years	------years		
1.8	Have you taken training of food safety?	1.	Yes     2. No		
Section II: Information on medical history of study participants
No.	Questions 	Classification and Category Code  	Remark	
2.1	 Have you done medical checkups in the last three months?	1. Yes    2. No		
2.2	Do you have any history of Admission to a hospital in the last three months?	1. Ye     2. No		
2.3	Do you have a history of medical instrumentation while on admission in the last three months?	1. Yes    2. No		
2.4	Do you have Used antibiotics without the consultation of a medical practitioner?	1. Yes
2. No		
2.5	Do you have a history of diarrhea in the last three months?	1. Yes  
  2. No		
2.6	Do have a history of urinary tract infection in the last three month?	1. Yes   
 2. No 		
2.7	Do you have Antibiotics used in the last 3 months?
	1.Yes
2. No		
2.8	Do have a history of chronic disease?	1. Yes    2. No 		
Section III: Information on hygiene-related factors
No.	Questions 	Classification and Category Code  	Remark	
3.1	  Do have frequent Hands washing habit?	1.Yes
2.No		
3.2	How do you wish your hands?	1. only with water
2. with soap		
3.3	Fingernail status	1. trimmed 
2. not trimmed		
3.4	Wear hair garment	1.	Yes
2.	No 		
3.5	Source of water for drinking?	1. pipe 
2. hand dug well		
3.6	 Do Have pit latrine toilet facilities?
	1. Yes
2. No		
3.7	Do you have a history of drinking unpasteurized milk?	1. Yes
2. No		
3.8	Do you have a history of eating raw meat?	1. Yes
2. No		
3.9	Do you have a history of eating raw vegetables?	1. Yes
2. No 		
