# Supplementary material for: High prevalence of fecal carriage of Extended-spectrum beta-lactamase and carbapenemase-producing Enterobacteriaceae among food handlers at the University of Gondar, Northwest Ethiopia
Source: PLoS One. 2022 Mar 17;17(3):e0264818. doi: 10.1371/journal.pone.0264818 (PMC8929611; doi:10.1371/journal.pone.0264818)
Supplement: S1 Protocol — Based on CLSI criteria, interpret the zones sizes of each antimicrobial, reporting the organism as ‘Resistant’, ‘Intermediate/Moderately susceptible’, ‘Susceptible see below the table’. (RTF) [file pone.0264818.s002.rtf]

S1 Protocol. The AST interpretation chart (extracted from CLSI, guideline 2020)
Based on CLSI criteria, interpret the zones sizes of each antimicrobial, reporting the organism as 'Resistant', 'Intermediate/Moderately susceptible', 'Susceptible see below the table'.
Sr.no	Drug Name	Susceptible	Intermediate	Resistance		Remark 	
1	Augmentin (Aug)	≥25	-	≤18			
2	Chloramphenicol (CHL)	≥18	13-17	≤12			
3	Ciprofloxacin (CIP)	≥26	22-25	≤21			
4	Tetracycline (TET)	≥15	12-14	≤11			
5	Ceftazidime (CAZ)	≥21	18-20	≤17			
6	Cefotaxime (CTX)	≥26	23-25	≤22			
7	Cotrimoxazole (SXT)	≥16	11-15	≤10			
8	Gentamycin  (GEN) 	≥15	13-14	≤12			
9	Meropenem (MER)	≥23	20-22	≤19			
10	Cefoxitin (CXT)	≥18	15-17	≤14			
11	Ceftriaxone  (CTR)	≥23	20-22	≤19			
