# Supplementary material for: High prevalence of fecal carriage of Extended-spectrum beta-lactamase and carbapenemase-producing Enterobacteriaceae among food handlers at the University of Gondar, Northwest Ethiopia
Source: PLoS One. 2022 Mar 17;17(3):e0264818. doi: 10.1371/journal.pone.0264818 (PMC8929611; doi:10.1371/journal.pone.0264818)
Supplement: S2 Protocol — (RTF) [file pone.0264818.s003.rtf]

Stool specimen collection 
The recovery of bacterial pathogens from fecal specimens will help confirm the diagnosis of bacterial pathogens as manifested by diarrhea and/or dysentery. 
1.	Obtain a collection kit containing Cary Blair transport media from this laboratory. 
·	NOTE: The enteric kits have an expiration date. If kits are expired, return them to this laboratory for replacement. 
2.	Collect feces from patients as soon after the onset of illness as possible, and before the start of treatment. Specimens may be obtained by covering the rear half of the toilet rim below the seat with plastic kitchen wrap to catch feces. 
3.	Transfer a sample (no more than one ounce) of the specimen using the spatula attached to the container lid into the Cary Blair medium supplied in the kit and mix thoroughly. Dispose of the unused specimen in the toilet, rinse the plastic in the toilet and dispose of it in the trash. 
4.	For liquid stool specimens, no more than 10 ml should be added to the Cary Blair medium and mixed. 
5.	Place the specimen and requisition form in the transport pouch and arrange for courier pickup. 
·	Specimens must be processed by the laboratory within 72 hours of collection. 
·	NOTE: Do not ship stool cultures without using the Cary Blair transport medium. 
 Culture of media preparation 
MacConkey (MAC) agar 
MacConkey Agar was used for the isolation and differentiation of lactose-no fermenting, gram-negative enteric bacteria from lactose-fermenting organisms. It is recommended that MAC medium be purchased commercially because preparing it with individual ingredients produces variability among lots. 
Media preparation 
1.	Prepare MAC according to the manufacturer's instructions. 
2.	Sterilize the medium by autoclaving at 121°C for 15 minutes. 
3.	Cool to 50°C.
4.	Dispense 20-25ml into 15x100 mm Petri dishes. Allow the media to solidify and condensation to dry. 
5.	Place the plates in sterile plastic bags and store at 4ºC until use.
Mueller-Hinton agar 
Mueller-Hinton agar was used for making the media required for susceptibility testing. It is recommended that the dehydrated Mueller-Hinton agar medium be purchased commercially because preparing it with individual ingredients can diminish the quality. 
Media Preparation 
1.	Follow the manufacturer's instructions to prepare MHA from a commercially available dehydrated base. 
2.	After autoclaving, cool the agar from 45°C to 50°C. 
3.	Pour agar into flat-bottom glass or plastic Petri dishes on a level pouring surface. 
	Measure 60-70 ml medium per plate into 15x150 mm plates or measure 25-30 ml per plate into 15x100 mm plates to give a uniform depth of approximately 4 mm. 
	Plates should be uniformly 3-4 mm thick as the rate of diffusion of the antimicrobial agents or the activity of the drugs may be affected.
	Using more or less agar will affect susceptibility results. 
4.	Allow the media to solidify and condensation to dry. 
5.	The pH of MHA should be 7.2-7.4. 
	Do not attempt to adjust the pH of the MHA test medium if it is outside the range. 
6.	Place the plates in sterile plastic bags and store it at 4ºC until use. 
 Laboratory activities
1.	Gram's stain preparation and Examinations 
Gram Staining was the type of differential staining which is common, important, and most used. This method differentiates the bacteria into Gram Positive, appears red or pink, appears purple, and Gram-Negative, which helps to differentiate and classify microorganisms.
Reagents Used and Materials Required
	Crystal Violet, the primary stain
	Iodine, the mordant
	A decolorize made of acetone and alcohol (95%)
	Safranin, the counterstain
	Clean glass slides
	Inoculating loop
	Bunsen burner
	Bibulous paper
	Microscope
	Immersion oil
	Tape water
	18 to 24-hour cultures of the organism
The procedure of Gram Staining
1.	the slides  were labeled clearly with the code number
2.	With a sterile cooled loop, place a loopful of the culture on the slide. 
3.	Spread using a circular motion of the inoculating loop to about one centimeter in diameter.
4.	Air dry and heat fix
5.	Place slide with a heat-fixed smear on staining tray.
6.	Gently flood smear with crystal violet and let stand for 1 minute.
7.	Tilt the slide slightly and gently rinse with tap water or distilled water using a wash bottle.
8.	Gently flood the smear with Gram's iodine and let stand for 1 minute.
9.	Tilt the slide slightly and gently rinse with tap water or distilled water using a wash bottle.
10.	The smear will appear as a purple circle on the slide.
11.	Decolorize using 95% acetone alcohol.
12.	Tilt the slide slightly and apply the alcohol drop by drop for 10 to 30 seconds until the alcohol runs almost clear.
13.	Immediately rinse with water.
14.	Gently flood with Safranin to counter-stain and let stand for 45 seconds.
15.	Tilt the slide slightly and gently rinse with tap water or distilled water using a wash bottle.
16.	Blot dry the slide with bibulous paper.
17.	Examine the smear using a light microscope under oil immersion.
18.	And report the result as follows.
         Gram-Positive: Purple Color
         Gram-Negative: Red/Pink Color
2.	Biochemical test
For the identification of Enterobacteriaceae, we had used the following biochemical tests medium.
v	Triple Sugar Iron: which has multi-purposes, helps to see fermentation of Carbohydrates, production of gas and H2S
v	(Indole)Tryptophan: helps to see the production of tryptophanase enzyme
v	Urea: shows the ability of the isolates to hydrolyze urea by producing the enzyme urease
v	Citrate: which is essential to see bacterial ability to utilize it as the sole carbon source
v	Motility: helps to see the ability of an organism to move by itself using propeller-like flagella
v	Lysine decarboxylase: lysine get decarboxylase when Enterobacteriaceae isolates produce Lysine decarboxylase enzyme
Procedure
1.	Prepare a 0.5 McFarland suspension of the test organism with nutrient broth.
2.	A loop full of the bacterial suspension is inoculated in (Indole) tryptophan, urea, and triple sugar iron, citrate, and lysine decarboxylase and motility tests medium.
3.	Incubate at 37Oc + 20C for 18-24 hours
4.	Look for production of Indole by adding Kovacs reagent in SIM medium
5.	Look for color change in the other test medium (turbidity for motility) of the medium
6.	Identify the test organism by following the standard biochemical test result chart for Enterobacteriaceae.
 Bacteriological and biochemical characteristics of isolated bacteria      
   Enterobacteriaceae (K. pneumoniae, E. coli, and E. cloacae..............etc.)
They are gram-negative rods, either motile or non-motile; grow well on MacConkey agar aerobically and anaerobically. It is catalase-positive and oxidase-negative, reducing nitrate to nitrite.
Table:  phenotypic and Biochemical characteristics of isolated bacteria
              Standard Biochemical tests for Gram-negative rods	
Bacterial isolates 	MAC	TSI
	Citrate 
test	urease
	
SIM	LDC	
		Slant	But	H2S	Gas			Indole	Motility 		
E. coli	LF	A	A	-	+	_	_	+	+	+	
K. pneumoniae	LF	A	A	-	+	+	+	_	_	+	
C. freundii	LF	A	A	+	+	+	+	_	+	_	
E. cloacae	LF	A	A	_	+	+	-	_	+	_	
Serratia species	LF	A	A	_	_	_	_	_	_	+	
Providencia species 	
NLF	K	A	_	+	+	+	+	+	_	
M. morganii	NLF	K	A	_	+	_	+	+	+	_	
P. mirabilis	NLF	K	A	+	+	+/_	+	_	+	+	
 Key: LF= lactose fermenter, NLF=non lactose fermenter, MAC=MacConkey agar, A= Acid, K= Alkaline, + = Positive, - = Negative, SIM=Sulfide-Indole-Motility, TSI=Triple sugar iron agar, LDC=lysine decarboxylase.
3.	Antimicrobial Susceptibility Testing
        Method: Modified Kirby-Bauer susceptibility testing technique
      Required materials: Mueller Hinton agar, Antimicrobial discs, McFarland machine, and Control strains to test the performance standard of Antimicrobial disks.
 Procedure:
1.	Using a sterile wire loop, 3 to 5 pure colony picked and prepare bacterial suspension equivalent with 0.5 McFarland standard in sterile 0.85% physiological saline.
2.	Using a sterile swab, inoculate a plate of Mueller Hinton agar. Streak the swab evenly over the surface of the medium in three directions, rotating the plate approximately 60o to ensure even distribution.
3.	With the petri dish lid in place, allow 3-5 minutes (no longer than 15 minutes) for the surface of the agar to dry.
4.	Using sterile forceps, place the appropriate antimicrobial discs,    evenly distributed on the inoculated plate.    Note: The disks should be about 15 mm from the edge of the plate and no closer than about 25 mm from disc to disc. No more than 6 discs should be applied (90 mm dish).  Each disc should be lightly pressed down to ensure its contact with the agar.  It should not be moved once in place.
5.	Within 30 minutes of applying the discs, invert the plate and incubate aerobically at 37 oC for 16-18 hours.
6.	After overnight incubation, examine the control and test plates to ensure the growth is confluent or near confluent. Using a ruler on the underside of the plate measures the diameter of each zone of inhibition in mm.
7.	Interpretation of zone sizes:
8.	Based on CLSI criteria, interpret the zones sizes of each antimicrobial, reporting the organism as 'Resistant', 'Intermediate/Moderately susceptible', 'Susceptible see below the table'.
Sr.no	Drug Name	Susceptible	Intermediate	Resistance		Remark 	
1	Augmentin (Aug)	≥25	-	≤18			
2	Chloramphenicol (CHL)	≥18	13-17	≤12			
3	Ciprofloxacin (CIP)	≥26	22-25	≤21			
4	Tetracycline (TET)	≥15	12-14	≤11			
5	Ceftazidime (CAZ)	≥21	18-20	≤17			
6	Cefotaxime (CTX)	≥26	23-25	≤22			
7	Cotrimoxazole (SXT)	≥16	11-15	≤10			
8	Gentamycin  (GEN) 	≥15	13-14	≤12			
9	Meropenem (MER)	≥23	20-22	≤19			
10	Cefoxitin (CXT)	≥18	15-17	≤14			
11	Ceftriaxone  (CTR)	≥23	20-22	≤19			
ESBL confirmation
Method: combined disk diffusion technique
    Required materials: suspected isolate, Muller Hinton agar, discs containing cephalosporin alone (cefotaxime, ceftazidime) and in combination with clavulanic acid and control strains to test the effectiveness of the disks.
   TEST PROCEDURE
1.	Using a fresh, pure culture prepare a suspension of the test organism equal to 0.5 McFarland Standard.
2.	Using a sterile cotton swab, spread the adjusted suspension over the entire area of a Mueller Hinton agar plate.
3.	Apply the discs onto the inoculated plate, ensuring sufficient space between individual discs to allow for proper measurement of inhibition zones.
4.	Incubate at 35±2°C for 18-24 hours.
5.	At the end of the incubation period, the inhibition zone around the cephalosporin disc combined with clavulanic acid is compared with the zone around the disc with the cephalosporin alone. The test is positive if the inhibition zone diameter is ≥ 5 mm larger with clavulanic acid than without.
4.	Modified Carbapenem Inactivation Methods (mCIM) for Suspected Carbapenemase Production in Enterobacteriaceae 
Purpose: mCIM is used for detecting carbapenemase in Enterobacteriaceae 
Reagent and materials 
	2 ml Trypticase soya broth (TSB)  
	Meropenem disks (10 ìg) 
	1-ìl inoculation loops 
	3 ml of Nutrient broth 
	Muller Hinton agar (MHA )plates (100 mm) 
	0.5 M EDTA
	Meropenem-susceptible indicator strain  E. coli (ATCC®a 25922) 
	Incubator 
	Ruler 
Procedure 
1.	For each isolate to be tested, emulsify a 1-ìL loopful of bacteria  with 2 ml of TSB for Enterobacteriaceae 
2.	Vortex for 10–15 seconds. 
3.	Add a 10-ìg Meropenem disk to each tube using sterile forceps. Ensure the entire disk is immersed in the suspension. 
4.	Incubate at 35°C ± 2°C in ambient air for 4 hours ± 15 minutes.
5.	Just before or immediately following completion of the TSB-Meropenem disk suspension incubation, prepare a 0.5 McFarland suspension (using the colony suspension method) of E. coli ATCC® 25922 in nutrient broth.
6.	Inoculate an MHA plate with E. coli ATCC® 25922 as for the routine disk diffusion procedure making sure the inoculum suspension preparation and MHA plate inoculation steps are each completed within 15 minutes. Allow the plates to dry for 3–10 minutes before adding the Meropenem disks.
7.	Remove the Meropenem disk from each TSB-Meropenem disk suspension using a 1ìL loop by placing the flat side of the loop against the flat edge of the disk and using surface tension to pull the disk out of the liquid. Carefully drag and press the loop along the inside edge of the tube to expel excess liquid from the disk. Continue using the loop to remove the disk from the tube and then place it on the MHA plate previously inoculated with the Meropenem-susceptible E. coli ATCC® 25922 indicator strain. Disk capacity: 4 disks on a 100 mm MHA plate.
8.	Invert and incubate the MHA plates at 35°C ± 2°C in ambient air for 18–24 hours.
9.	Following incubation, measure the zones of inhibition as for the routine disk diffusion method 
Test interpretation.
Carbapenemase was positive if zone diameter of 6–15 mm or presence of pinpoint colonies within a 16–18 mm zone.
·	If the test isolate produces a carbapenemase, the Meropenem in the disk will be hydrolyzed and there will be no inhibition or limited growth inhibition of the meropenem-susceptible E. coli ATCC® 25922.
·	Carbapenemase negative if zone diameter of ≥ 19 mm (clear zone) .If the test isolate does not produce carbapenemase, the meropenem in the disk will not be hydrolyzed and will inhibit the growth of the meropenem-susceptible E. coli ATCC® 25922.
